# Draft genome sequence of the moderately halophilic bacterium *Vreelandella alkaliphila* strain WG10-512 isolated from a Himalayan salt mine in India

**DOI:** 10.1128/mra.00737-26

**Published:** 2026-10-06

**Authors:** Abhishek Walia, Partiksha Rana, Anila Badiyal, Shailender K. Verma

**Affiliations:** 1 Department of Microbiology, College of Basic Sciences, CSK Himachal Pradesh Krishi Vishvavidyalaya 75210 , Palampur, Himachal Pradesh, India; 2 Department of Environmental Studies, University of Delhi 28742, Delhi, India; University of Wisconsin-Madison, Madison, Wisconsin, USA

**Keywords:** *Vreelandella alkaliphila*, moderately halophilic bacterium, whole-genome sequence, Himalayan salt mine

## Abstract

*Vreelandella alkaliphila* strain WG10-512, isolated from Gumma salt mines, India, was sequenced via Illumina NovaSeq. The 183-contig draft genome contains 3,435 genes with 99.93% completeness and 0.41% contamination. Taxonomic analysis confirmed its classification with 98.94% ANI, providing a valuable resource for studying microbial adaptation in saline environments.

## ANNOUNCEMENT

Members of the genus *Vreelandella* are moderately halophilic bacteria belonging to the family Halomonadaceae and are commonly associated with saline and hypersaline environments (1). These microorganisms possess diverse physiological adaptations that enable survival under osmotic stress and have attracted attention because of their ecological significance and potential applications in biotechnology and agriculture (2). Rather than making a general claim that genomic information on *Vreelandella* is limited, this study reports the genome sequence of a strain specifically isolated from saline water surrounding salt mines in the Himalayas (Table 1).

**TABLE 1:** Genome features of *V. alkaliphila* strain WG10-512 isolated from saline water surrounding the salt mines of the Himalayas in India^*b*^

Features	Value
Number of contigs	183
Genome size (bp)	3,605,406
GC content (%)	52.78
Total genes	3,435
Protein-coding genes	3,352
CDSs	3,372
Pseudogenes	20
Total RNA genes	63
tRNA genes	56
rRNA genes	3
16S rRNA	1
23S rRNA	2
5S rRNA	0
tmRNA	1
ncRNA genes	3
Genome completeness (%)	99.93
Contamination (%)	0.41
GTDB-Tk ANI (%)	98.94
16S rRNA identity (%)	100 (98% query coverage; 1,500 bp)
COG assignments^*a*^	2,991
GO terms^*a*^	67
KEGG orthologs^*a*^	2,141
CAZy families^*a*^	25

^*a*^ COG, GO, KEGG, and CAZy statistics were generated from the Bakta v1.9.4 and eggNOG-mapper v2.1.11 annotations rather than the PGAP annotation. The corresponding annotation files are publicly available in Zenodo (https://doi.org/10.5281/zenodo.21998665).

^*b*^ GO, gene ontology.

*Vreelandella alkaliphila* strain WG10-512 was isolated from a water sample collected aseptically from the salt mine region of Gumma (76.8534° E, 31.9672° N), Mandi District, Himachal Pradesh, India. The water sample was processed for the isolation of halophilic bacteria by culturing on nutrient agar (per liter: peptic digest of animal tissue, 5.0 g; sodium chloride, 5.0 g; beef extract, 1.5 g; yeast extract, 1.5 g; agar, 15.0 g; pH 8.0 ± 0.2 at 25°C) supplemented with 5%, 10%, and 15% (wt/vol) NaCl. Plates were incubated aerobically at 30°C for 72 h, and individual colonies obtained at 10% NaCl were purified by repeated streaking to obtain a pure culture, which was maintained for genomic characterization.

Genomic DNA was extracted from a pure culture using the DNeasy UltraClean Microbial Kit (Qiagen). DNA quality and quantity were assessed using NanoDrop spectrophotometry and agarose gel electrophoresis. Whole-genome shotgun sequencing was performed on the Illumina NovaSeq platform using paired-end 150-bp chemistry. Sequencing generated 12,686,996 raw reads corresponding to 1.903 Gb of sequence data. Following quality filtering, 12,473,000 reads and 1.847 Gb of high-quality sequence data were retained for downstream analyses. Raw reads were quality filtered using fastp v0.23.4 (3) to remove adapter sequences and low-quality reads, and the quality-filtered reads were used for *de novo* genome assembly with Unicycler v0.4.4 (4), followed by scaffolding using RagTag v2.1.0 (5). Genome quality was assessed using CheckM2 v1.0.1 (6); taxonomic assignment was performed using GTDB-Tk v2.4.0 (7); and assembly quality was evaluated using QUAST v5.0.2 (8). The deposited genome was annotated using the NCBI Prokaryotic Genome Annotation Pipeline (PGAP) (9), and additional structural and functional annotations were generated using Bakta v1.9.4 (10) and eggNOG-mapper v2.1.11 (11). Unless otherwise stated, default parameters were used for all software.

Taxonomic identification was evaluated using GTDB-Tk based on skani (12), and the 16S rRNA gene was extracted using Barrnap v0.9 (13) and compared using BLASTN against the 16S rRNA database. GTDB-Tk assigned WG10-512 to *Vreelandella alkaliphila* with an ANI of 98.94%, while 16S rRNA analysis showed 100% sequence identity over 98% query coverage (1,500 bp). These complementary analyses supported the taxonomic assignment of WG10-512 to *V. alkaliphila*.

The draft genome assembly consisted of 183 contigs with a total length of 3,605,406 bp and 52.78% GC content and was sequenced to an average coverage depth of approximately 465×. Genome annotation was performed using the NCBI PGAP, which identified 3,435 total genes and 3,372 CDSs, including 3,352 protein-coding genes and 63 RNA genes. The RNA complement included 56 tRNAs and 3 ncRNAs, and 20 pseudogenes were identified. Genome completeness was 99.93% and contamination was 0.41%. Additional structural and functional annotations were generated using Bakta v1.9.4 and eggNOG-mapper v2.1.11, which identified 2,991 COG assignments, 67 Gene Ontology terms, 2,141 KEGG orthologs, and 25 carbohydrate-active enzyme families. These additional annotation files have been deposited in Zenodo (DOI: https://doi.org/10.5281/zenodo.21998665).

**Fig 1:**
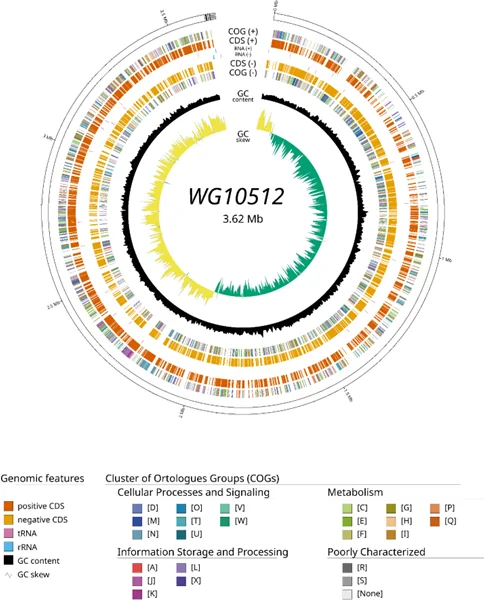
Circular genome map of *Vreelandella alkaliphila* strain WG10-512. From the outside inward, the rings represent contigs, coding sequences on the forward strand, tRNA and rRNA genes, coding sequences on the reverse strand, GC content, and GC skew. The figure was generated using GenoVi v0.4.3 (14).

The draft genome sequence of *V. alkaliphila* strain WG10-512 will facilitate future studies on microbial adaptation to saline conditions and the potential development of moderately halophilic microbial resources for sustainable management of salt-affected soils (Fig. 1).

## Data Availability

This Whole Genome Shotgun project has been deposited at DDBJ/ENA/GenBank under the accession number JBYRTC000000000. The version described in this paper is JBYRTC010000000. The genome sequence is associated with BioProject accession number PRJNA1469997 and BioSample accession number SAMN60394157. Additional genome annotation files generated using Bakta v1.9.4, eggNOG-mapper v2.1.11, and CAZyme analyses have been deposited in Zenodo (https://doi.org/10.5281/zenodo.21998665).
